# Serum Lipid Profile, Atherogenic Indices, and Systemic Inflammatory Markers in Retinal Vein Occlusion: A Retrospective Case–Control Study

**DOI:** 10.1155/joph/6862613

**Published:** 2026-10-15

**Authors:** Nurcan Gürsoy, Şenay Deniz

**Affiliations:** ^1^ Department of Ophthalmology, Of State Hospital Trabzon Türkiye; ^2^ Department of Ophthalmology Kuşadası State Hospital Aydın Türkiye

**Keywords:** atherogenic indices, case–control study, dyslipidemia, inflammatory markers, neutrophil-to-lymphocyte ratio, non-HDL cholesterol, retinal vein occlusion

## Abstract

**Purpose:**

Retinal vein occlusion (RVO) is a common retinal vascular disorder associated with systemic vascular and metabolic risk factors. Although both dyslipidemia and inflammation have been implicated in its pathogenesis, the associations of conventional lipid parameters, derived atherogenic indices, and hemogram‐derived inflammatory markers with RVO remain incompletely characterized.

**Methods:**

This retrospective, single‐center, case–control study included 81 patients with RVO and 91 controls without RVO. Hospital records from January 2025 through December 2025 were reviewed. Conventional lipid parameters, derived atherogenic indices, and systemic inflammatory markers were compared between groups. Multivariable logistic regression analyses were performed using separate models for conventional lipid parameters, derived lipid indices, and inflammatory indices to reduce collinearity and improve interpretability.

**Results:**

Patients with RVO were older than controls and had significantly higher monocyte, neutrophil, and leukocyte counts, as well as higher C‐reactive protein (CRP), low‐density lipoprotein cholesterol (LDL‐C), triglyceride, total cholesterol, and non–high‐density lipoprotein cholesterol (non‐HDL‐C) levels. Among the derived indices, total cholesterol/high‐density lipoprotein cholesterol (HDL‐C), LDL‐C/HDL‐C, and non‐HDL‐C/HDL‐C ratios were significantly higher in the RVO group, whereas neutrophil‐to‐lymphocyte ratio (NLR), platelet‐to‐lymphocyte ratio (PLR), neutrophil‐to‐platelet ratio (NTR), hemoglobin‐albumin‐lymphocyte‐platelet (HALP) score, systemic immune‐inflammation index (SII), and triglyceride/HDL‐C ratio were comparable between groups. In models adjusted for age, sex, and CRP, LDL‐C, non‐HDL‐C, and total cholesterol were associated with RVO, whereas triglyceride was not. Total cholesterol/HDL‐C, LDL‐C/HDL‐C, and non‐HDL‐C/HDL‐C ratios were also associated with RVO in models adjusted for age, sex, and CRP, whereas triglyceride/HDL‐C was not. None of the evaluated inflammatory indices was associated with RVO in models adjusted for age and sex.

**Conclusions:**

In this dataset, several cholesterol‐related variables were associated with RVO in models adjusted for age, sex, and CRP, whereas none of the evaluated hemogram‐derived inflammatory indices was associated with RVO in models adjusted for age and sex. Because these variables were examined in separate models with different covariate adjustments, the findings do not establish the superiority or greater relative importance of lipid markers.

## 1. Introduction

Retinal vein occlusion (RVO) is one of the most common retinal vascular disorders after diabetic retinopathy and remains an important cause of visual impairment [1, 2]. Although it presents as an ocular vascular event, RVO is increasingly understood as a multifactorial disease linked to systemic vascular dysregulation, aging, and cardiometabolic risk factors such as hypertension, diabetes mellitus, and dyslipidemia [2–4]. Current evidence also implicates thrombosis, endothelial dysfunction, and inflammatory activation in its pathogenesis [1, 5].

Because these mechanisms extend beyond the eye, readily available systemic biomarkers have attracted growing interest in RVO research [6]. Hemogram‐derived inflammatory indices are particularly appealing because they are inexpensive, routinely measured, and may reflect different components of low‐grade thromboinflammatory activity [7, 8]. Neutrophil‐to‐lymphocyte ratio (NLR) reflects the balance between neutrophil‐mediated inflammation and lymphocyte‐related immune regulation, whereas platelet‐to‐lymphocyte ratio (PLR) integrates platelet activation with lymphocyte‐mediated inflammatory status. Neutrophil‐to‐platelet ratio (NTR) may provide an additional estimate of the relationship between neutrophil‐driven inflammation and platelet‐related thrombotic activity. Systemic immune‐inflammation index (SII) combines neutrophil, platelet, and lymphocyte counts and may therefore represent a broader index of systemic immune‐inflammatory activation. Hemoglobin‐albumin‐lymphocyte‐platelet (HALP) score integrates hemoglobin, albumin, lymphocyte count, and platelet count and has been proposed as a composite marker of immunonutritional and inflammatory status [9]. In a systematic review and meta‐analysis, both NLR and PLR were significantly higher in patients with RVO than in controls, supporting a potential association between systemic inflammation and RVO [10].

Lipid‐related abnormalities are of particular interest in RVO because RVO develops in a microvascular setting in which conventional lipid fractions may not fully reflect the overall atherogenic milieu [11, 12]. Nontraditional lipid measures may therefore be especially relevant, as calculated lipid ratios have been proposed to reflect the balance between atherogenic and antiatherogenic lipoprotein fractions more comprehensively than isolated lipid values [11]. Total cholesterol/HDL and LDL/HDL ratios express the relationship between cholesterol‐rich atherogenic particles and HDL‐related protective capacity, whereas triglyceride/HDL ratio is commonly considered a marker related to insulin resistance, triglyceride‐rich lipoproteins, and small dense LDL patterns. Non‐HDL/HDL ratio may further reflect the balance between the total atherogenic cholesterol burden and HDL‐associated antiatherogenic activity [12–14]. Although most of this evidence derives from systemic vascular rather than RVO‐specific research, lipid‐related pathways, especially HDL‐associated mechanisms, also appear relevant to microvascular occlusive phenotypes, supporting the evaluation of both conventional lipid parameters and derived atherogenic indices in RVO [11, 15].

Accordingly, the present study aimed to compare conventional serum lipid parameters, derived atherogenic indices, and systemic inflammatory markers between patients with RVO and individuals without RVO, and to evaluate their associations with RVO after adjustment for the covariates available in the dataset.

## 2. Materials and Methods

### 2.1. Study Design and Setting

This was a retrospective, single‐center, case–control study conducted at the Department of Ophthalmology, Erzincan Mengücek Gazi Training and Research Hospital, Türkiye. The study compared conventional serum lipid parameters, derived atherogenic indices, and systemic inflammatory markers between patients with RVO and individuals without RVO. Hospital records from January 2025 through December 2025 were retrospectively reviewed.

### 2.2. Participants

Adults aged 18 years or older were eligible for inclusion. The RVO group consisted of patients diagnosed with RVO in the ophthalmology outpatient clinic on the basis of clinical examination and/or retinal imaging and who had available complete blood count and biochemical test results obtained within the same day. The control group consisted of hospital‐based ophthalmic controls evaluated during the same study period. Controls were adults without a diagnosis or documented history of RVO who had complete blood count and biochemical test results available in the hospital records. They were mainly individuals undergoing cataract assessment, presbyopia‐related examination, routine outpatient ophthalmology follow‐up, or preoperative evaluation. The laboratory tests were generally obtained as part of routine clinical or preoperative care, although the precise indication for testing was not consistently documented in the structured records. Controls were not recruited as healthy community participants. Stable chronic vascular and metabolic conditions were not exclusion criteria; however, information on hypertension, diabetes mellitus, smoking status, body mass index, cardiovascular or renal disease, and lipid‐lowering treatment was not consistently available as structured data and therefore could not be reliably compared between the groups. The same exclusion criteria were applied to both groups. Individuals with acute infection, active inflammatory disease, autoimmune disease, active malignancy, recent chemotherapy or radiotherapy, systemic corticosteroid or immunosuppressive treatment, or acute renal or hepatic failure were excluded because these conditions could substantially affect inflammatory or biochemical biomarkers. All eligible records meeting these criteria during the study period were included in the final analysis.

### 2.3. Data Collection and Variables

Data were extracted from the hospital information system and entered into an anonymized dataset. The recorded variables included age, sex, hemoglobin, platelet count, lymphocyte count, monocyte count, neutrophil count, total leukocyte count, albumin, C‐reactive protein (CRP), high‐density lipoprotein cholesterol (HDL‐C), low‐density lipoprotein cholesterol (LDL‐C), triglycerides, total cholesterol, and non‐HDL cholesterol. In addition to these conventional parameters, the following inflammatory and atherogenic indices were calculated from the recorded laboratory values: NLR, PLR, NTR, HALP score [HALP = hemoglobin (g/L) × albumin (g/L) × lymphocyte count/platelet count], systemic immune‐inflammation index (SII = neutrophil × platelet/lymphocyte), total cholesterol/HDL ratio, LDL/HDL ratio, triglyceride/HDL ratio, and non‐HDL/HDL ratio. For HALP calculation, hemoglobin values recorded in g/dL were converted to g/L to maintain consistency with the standard formula. When available, RVO subtype information was extracted from the structured ophthalmology records. Because subtype information was unavailable for most cases, RVO was analyzed as an aggregate clinical category. The available subtype data were reported descriptively, and no subtype‐specific statistical analyses were performed.

### 2.4. Ethics Approval

Ethical approval was obtained from the Erzincan Binali Yıldırım University Non‐Interventional Clinical Research Ethics Committee (meeting no. 005, decision no. 2026–05/06, dated 05 March 2026). The study was conducted in accordance with the Declaration of Helsinki. Given the retrospective design and the use of anonymized routine clinical data, informed consent was not required under local ethics procedures.

### 2.5. Statistical Analysis

Statistical analyses were performed using IBM SPSS Statistics. Continuous variables were evaluated for distributional characteristics using visual inspection of histograms and Q–Q plots, together with the Shapiro–Wilk test. Data were summarized as mean ± standard deviation for approximately normally distributed variables and as median (interquartile range) for nonnormally distributed variables, whereas categorical variables were expressed as number and percentage. Between‐group comparisons were performed using the independent‐samples *t* test for normally distributed continuous variables and the Mann–Whitney *U* test for nonnormally distributed continuous variables. Categorical variables were compared using Pearson’s chi‐square test. Because age is an established risk factor for RVO and differed between the study groups, age was included as an adjustment variable in all multivariable logistic regression models. Because conventional lipid parameters, derived lipid ratios, and inflammatory indices were biologically and mathematically related, separate multivariable models were constructed to reduce collinearity and improve interpretability. Conventional lipid models were adjusted for age, sex, and CRP, with LDL‐C, non‐HDL cholesterol, triglycerides, and total cholesterol entered separately. Derived lipid index models were also adjusted for age, sex, and CRP, with total cholesterol/HDL, LDL/HDL, triglyceride/HDL, and non‐HDL/HDL ratios evaluated in separate models. Inflammatory index models were adjusted for age and sex, with NLR, PLR, NTR, HALP, and SII entered separately. Odds ratios (ORs) with 95% confidence intervals (95% CIs) were reported. Model fit was assessed using the Hosmer–Lemeshow goodness‐of‐fit test, and model performance was additionally described by Nagelkerke *R* ^2^ and overall classification accuracy. Only participants with complete laboratory data for the variables included in the primary analyses were included in the final dataset. No missing data were present for the demographic or laboratory variables analyzed; therefore, no imputation was performed. Because hypertension, diabetes mellitus, smoking status, body mass index, cardiovascular or renal disease, and lipid‐lowering treatment were not consistently available as structured variables, they could not be reliably incorporated into the multivariable models. A two‐sided *p* value of < 0.05 was considered statistically significant.

## 3. Results

A total of 172 participants were included in the study, comprising 81 patients with RVO and 91 controls. RVO subtype information was documented in 18 of the 81 RVO cases; among these, 14 were classified as BRVO and 4 as CRVO, while no documented HRVO case was identified. Accordingly, all subsequent analyses treated RVO as an aggregate clinical category. Overall, 94 participants (54.7%) were women and 78 (45.3%) were men. Sex distribution was comparable between the groups (48.1% vs. 60.4% women in the RVO and control groups, respectively; Pearson’s *χ* ^2^ = 2.612, *p* = 0.106), whereas the RVO group was significantly older than the control group [median 70 (IQR 11) vs. 67 (IQR 11) years; *p* = 0.003]. This age imbalance was considered in the interpretation of the results, and age was included as an adjustment variable in all multivariable models. Conventional hematologic, inflammatory, and lipid parameters are presented in Table 1. Compared with controls, patients with RVO had significantly higher monocyte, neutrophil, and leukocyte counts, together with higher CRP, LDL cholesterol, triglyceride, total cholesterol, and non‐HDL cholesterol levels. In contrast, hemoglobin, platelet count, lymphocyte count, albumin, and HDL cholesterol levels did not differ significantly between the groups.

**TABLE 1:** Demographic, hematologic, and conventional biochemical/lipid parameters in the RVO and control groups.

Variable	RVO group (*n* = 81)	Control group (*n* = 91)	Statistic	*p* value
Documented RVO subtype				
BRVO	14 (17.2)			
CRVO	4 (4.9)			
Subtype not documented	63 (77.8)			
Age (years)	70 (11)	67 (11)	*U* = 2703.5	0.003
Female sex, *n* (%)	39 (48.1)	55 (60.4)	*χ* ^2^ = 2.612	0.106
Hemoglobin (g/dL)	14.00 ± 1.60	14.12 ± 1.46	*t* = −0.544	0.587
Platelet count (× 10^9^/L)	257 (51)	252 (89)	*U* = 3504.5	0.579
Lymphocyte count (× 10^9^/L)	2.53 (1.14)	2.29 (0.78)	*U* = 3265.5	0.198
Monocyte count (× 10^9^/L)	0.59 (0.25)	0.50 (0.20)	*U* = 2953.5	0.025
Neutrophil count (× 10^9^/L)	4.43 (1.81)	3.82 (1.39)	*U* = 2928.0	0.020
Leukocyte count (× 10^9^/L)	7.66 (3.16)	6.80 (1.99)	*U* = 2748.5	0.004
Albumin (g/L)	41.10 (4.75)	41.50 (3.00)	*U* = 3259.0	0.191
C‐reactive protein (mg/L)	4.50 (5.06)	3.17 (0.41)	*U* = 1573.5	< 0.001
HDL cholesterol (mg/dL)	50.0 (12.8)	50.0 (12.0)	*U* = 3579.5	0.745
LDL cholesterol (mg/dL)	128.98 ± 38.21	117.57 ± 29.55	*t* = 2.170^∗^	0.032
Triglycerides (mg/dL)	132 (95)	114 (63)	*U* = 3030.0	0.044
Total cholesterol (mg/dL)	206 (65)	187 (46)	*U* = 2996.0	0.034
Non‐HDL cholesterol (mg/dL)	156.90 (54.85)	138.00 (47.00)	*U* = 2893.5	0.015

*Note:* Data are presented as *n* (%), mean ± standard deviation, or median (interquartile range), as appropriate. Pearson’s chi‐square test was used for categorical variables. Normally distributed continuous variables were compared using the independent‐samples *t* test; non‐normally distributed variables were compared using the Mann–Whitney *U* test.

^∗^Welch *t*‐test value was reported for LDL because homogeneity of variances was not met.

Derived inflammatory and atherogenic indices are summarized in Table 2. Among these, total cholesterol/HDL, LDL/HDL, and non‐HDL/HDL ratios were significantly higher in the RVO group, whereas NLR, PLR, NTR, HALP score, SII, and triglyceride/HDL ratio were comparable between groups.

**TABLE 2:** Derived inflammatory and atherogenic indices in the RVO and control groups.

Variable	RVO group (*n* = 81)	Control group (*n* = 91)	Statistic	*p* value
Neutrophil‐to‐lymphocyte ratio (NLR)	1.73 (1.01)	1.76 (0.79)	*U* = 3329.5	0.275
Platelet‐to‐lymphocyte ratio (PLR)	106.08 (45.44)	113.17 (47.38)	*U* = 3649.5	0.912
Neutrophil‐to‐platelet ratio (NTR)	0.0171 (0.01)	0.0157 (0.01)	*U* = 3229.0	0.161
HALP score	52.44 (29.86)	52.80 (21.22)	*U* = 3565.0	0.712
Systemic immune‐inflammation index (SII)	458.94 (304.77)	449.08 (267.15)	*U* = 3274.0	0.207
Total cholesterol/HDL ratio	4.26 ± 1.03	3.92 ± 0.87	*t* = 2.335	0.021
LDL/HDL ratio	2.65 ± 0.80	2.40 ± 0.71	*t* = 2.176	0.031
Triglyceride/HDL ratio	2.77 (2.29)	2.32 (1.52)	*U* = 3111.5	0.078
Non‐HDL/HDL ratio	3.26 ± 1.03	2.92 ± 0.87	*t* = 2.335	0.021

*Note:* Data are presented as mean ± standard deviation or median (interquartile range), as appropriate. Normally distributed variables were compared using the independent‐samples *t* test, and nonnormally distributed variables were compared using the Mann–Whitney *U* test.

Multivariable logistic regression analyses are shown in Tables 3, 4, and 5. In models adjusted for age, sex, and CRP, LDL cholesterol, non‐HDL cholesterol, and total cholesterol were associated with RVO, whereas triglyceride was not. Among the conventional lipid models, the model containing non‐HDL cholesterol had the highest Nagelkerke *R* ^2^, whereas the total cholesterol model had the highest overall classification accuracy. These model characteristics are reported descriptively and should not be interpreted as establishing the relative importance or superiority of individual markers.

**TABLE 3:** Multivariable logistic regression models for conventional lipid parameters and RVO.

Model	Main predictor	Adjusted OR	95% CI	*p* value	Nagelkerke *R* ^2^	Hosmer–Lemeshow p	Overall correct (%)
Model 1	LDL cholesterol	1.014	1.003–1.025	0.011	0.285	0.345	69.8
Model 2	Non‐HDL cholesterol	1.012	1.003–1.020	0.007	0.291	0.735	72.1
Model 3	Triglyceride	1.004	0.999–1.009	0.090	0.260	0.301	69.8
Model 4	Total cholesterol	1.011	1.002–1.019	0.010	0.286	0.577	72.7

*Note:* All models were adjusted for age, sex, and CRP. Age remained significant in all models, whereas sex was not significant.

**TABLE 4:** Age‐ and sex‐adjusted logistic regression models for inflammatory indices and RVO.

Model	Main predictor	Adjusted OR	95% CI	*p* value	Nagelkerke *R* ^2^	Hosmer–Lemeshow p	Overall correct (%)
NLR model	NLR	1.323	0.920–1.902	0.131	0.103	0.550	59.3
PLR model	PLR	1.003	0.997–1.010	0.337	0.091	0.280	61.0
NTR model	NTR	unstable^∗^	unstable^∗^	0.465	0.088	0.100	62.2
HALP model	HALP	0.997	0.984–1.011	0.671	0.085	0.106	60.5
SII model	SII	1.001	1.000–1.002	0.110	0.104	0.790	58.7

*Note:* All models were adjusted for age and sex. Age remained significant in all models, whereas sex was not significant.

^∗^The NTR estimate was unstable because of scaling/distribution and should be interpreted with caution.

**TABLE 5:** Multivariable logistic regression models for derived lipid indices and RVO.

Model	Main predictor	Adjusted OR	95% CI	*p* value	Nagelkerke *R* ^2^	Hosmer–Lemeshow p	Overall correct (%)
Total cholesterol/HDL model	Total cholesterol/HDL	1.562	1.085–2.248	0.016	0.280	0.105	70.3
LDL/HDL model	LDL/HDL	1.656	1.046–2.624	0.031	0.272	0.152	69.2
TG/HDL model	TG/HDL	1.182	0.965–1.449	0.106	0.259	0.327	69.8
Non‐HDL/HDL model	Non‐HDL/HDL	1.562	1.085–2.248	0.016	0.280	0.105	70.3

*Note:* All models were adjusted for age, sex, and CRP. Age and CRP remained significant in all models, whereas sex was not significant. Because total cholesterol/HDL and non‐HDL/HDL are mathematically related, these two models yielded identical fit statistics.

In models adjusted for age and sex, none of the inflammatory indices, including NLR, PLR, NTR, HALP, and SII, was associated with RVO. Among the derived lipid indices, total cholesterol/HDL, LDL/HDL, and non‐HDL/HDL ratios were associated with RVO in models adjusted for age, sex, and CRP, whereas the triglyceride/HDL ratio was not.

## 4. Discussion

The present study showed that patients with RVO had a more atherogenic lipid profile than the hospital‐based ophthalmic controls, characterized by higher LDL cholesterol, non‐HDL cholesterol, total cholesterol, and cholesterol‐based lipid ratios. In models adjusted for age, sex, and CRP, LDL cholesterol, non‐HDL cholesterol, total cholesterol, total cholesterol/HDL, LDL/HDL, and non‐HDL/HDL were associated with RVO, whereas triglyceride and triglyceride/HDL ratio were not. In contrast, the evaluated hemogram‐derived inflammatory indices, including NLR, PLR, NTR, HALP, and SII, were not associated with RVO in models adjusted for age and sex. These findings must be interpreted in light of the covariates available in the retrospective dataset. The RVO group was significantly older than the control group, and although age was included in all multivariable models, residual age‐related confounding cannot be excluded. More importantly, hypertension, diabetes mellitus, smoking status, body mass index, cardiovascular or renal disease, and lipid‐lowering treatment could not be incorporated into the models. The observed associations therefore cannot be considered independent of established vascular and metabolic risk factors. In addition, because subtype‐specific data were incomplete, the findings should not be assumed to apply equally to BRVO, CRVO, and HRVO [1–4].

The lipid‐related findings are biologically plausible in light of the current understanding of RVO pathophysiology. Endothelial dysfunction, thrombosis, and impaired microvascular perfusion are central to RVO, and lipid‐related vascular injury may reasonably contribute to this process [4, 16]. In our cohort, cholesterol‐centered variables showed statistically significant associations with RVO, whereas triglyceride‐based measures did not. This pattern is descriptive and does not establish the relative importance of cholesterol‐ and triglyceride‐related pathways. In the broader vascular literature, calculated lipid indices have been proposed to provide additional information beyond the conventional lipid profile, and non‐HDL cholesterol has been emphasized as a clinically relevant marker of atherogenic burden [11, 13, 17–19]. The reported associations of non‐HDL‐related indices with small dense LDL‐related risk provide biological context for evaluating these measures in retinal microvascular occlusive disease [12, 14, 20].

Among the conventional lipid models, the model containing non‐HDL cholesterol had the highest Nagelkerke *R* ^2^, whereas the total cholesterol model had the highest overall classification accuracy. These model characteristics are reported descriptively and should not be interpreted as evidence that either marker is superior to, or relatively more important than, the other evaluated markers. Nevertheless, because non‐HDL cholesterol encompasses all atherogenic lipoproteins, including LDL and triglyceride‐rich remnants, it may reflect cumulative atherogenic burden [13, 21]. This may be especially relevant in RVO, where multiple overlapping vascular mechanisms likely contribute to venous occlusion [4, 22]. The associations observed for total cholesterol/HDL, LDL/HDL, and non‐HDL/HDL ratios in models adjusted for the available covariates support further investigation of composite lipid measures in RVO. However, because important vascular and metabolic confounders were unavailable, these results should be interpreted as exploratory associations rather than as evidence that the evaluated lipid ratios have effects independent of established RVO risk factors [23]. Together, these results suggest that conventional cholesterol‐related parameters and derived atherogenic ratios may contribute to systemic profiling in patients with RVO. However, given the retrospective design, these markers should be interpreted as associated laboratory features rather than as indicators with established predictive utility [11, 13, 17, 18].

By contrast, the inflammatory findings were more conservative than might be expected from some previous studies. A systematic review and meta‐analysis reported significantly higher NLR and PLR values in patients with RVO, and individual retrospective studies have also suggested that blood count–derived inflammatory indices may differ between patients with RVO and controls [7, 10, 24]. In the present study, however, none of the evaluated hemogram‐derived inflammatory indices was significantly associated with RVO in models adjusted for age and sex. Several explanations are possible. First, the control group consisted of hospital‐based ophthalmic controls rather than healthy community controls. Some controls may therefore have had stable systemic vascular or metabolic conditions and a degree of baseline low‐grade inflammation, as suggested by the relatively elevated CRP level in the control group. This may have attenuated between‐group differences in hemogram‐derived inflammatory indices. Second, these indices are nonspecific and may be influenced by age, comorbid vascular disease, physiological variation, and the timing of blood sampling relative to the acute occlusive event [25]. Third, the absence of statistically significant associations in the age‐ and sex‐adjusted models does not exclude a role for systemic inflammation in RVO, particularly because several important vascular and metabolic covariates were unavailable and could not be incorporated into the analyses. Fourth, inflammatory activity in RVO may involve local retinal and vascular mechanisms that are not adequately reflected by stable changes in peripheral blood cell ratios [4]. In this context, the statistically significant association of CRP in the lipid models suggests that broader systemic inflammatory activity may still be relevant, although the present study does not establish the relative importance or incremental value of CRP compared with hemogram‐derived inflammatory indices [4, 6, 7, 10].

From a clinical perspective, the present findings should be interpreted cautiously. In this dataset, statistically significant associations were observed for several cholesterol‐related variables, whereas none of the evaluated hemogram‐derived inflammatory indices was associated with RVO. However, because these variables were evaluated in separate models with different covariate adjustments, this pattern does not establish the superiority or greater relative importance of lipid markers. The retrospective case–control design and the absence of adjustment for several chronic vascular and metabolic confounders further limit the clinical interpretation of the findings. In particular, hypertension, diabetes mellitus, smoking status, body mass index, cardiovascular or renal disease, and lipid‐lowering treatment may be associated with both dyslipidemia and RVO, and residual confounding is therefore likely. These findings do not indicate that lipid markers can predict RVO or guide management independently. Rather, routine lipid parameters may contribute to the broader systemic assessment of patients with RVO when interpreted alongside age, vascular risk factors, ocular findings, and the overall clinical context.

### 4.1. Limitations

Several limitations should be acknowledged. First, the retrospective, single‐center, case–control design limits causal inference and generalizability. Second, the control group consisted of hospital‐based ophthalmic patients rather than healthy community participants. Some controls may have had stable chronic vascular or metabolic conditions that were not consistently available in the structured records. This may have introduced selection bias, attenuated differences in inflammatory markers, and influenced lipid comparisons. Accordingly, the findings may not be generalizable to comparisons with healthy community populations. Third, several chronic vascular and metabolic covariates, including hypertension, diabetes mellitus, smoking status, body mass index, cardiovascular or renal disease, and lipid‐lowering treatment, were not consistently available as structured variables and therefore could not be included in the multivariable models. Consequently, substantial residual confounding cannot be excluded, particularly for lipid‐related associations, and the reported findings cannot be considered independent of these established vascular and metabolic risk factors. Fourth, RVO subtype was documented in only 18 of 81 cases, which precluded reliable BRVO‐, CRVO‐, or HRVO‐specific analyses. Therefore, RVO was analyzed as an aggregate clinical category, and the findings should not be assumed to apply equally to individual RVO subtypes. Fifth, all laboratory parameters were derived from routine clinical records, and the temporal relationship between biomarker levels and RVO onset could not be standardized. Finally, more specific lipid‐related markers, such as apolipoprotein B, lipoprotein (a), and directly measured small dense LDL, were not available. Accordingly, the findings should be interpreted as associations based on conventional and calculated lipid measures rather than as causal, predictive, independent, or subtype‐specific evidence.

## 5. Conclusion

In conclusion, several cholesterol‐related variables were associated with RVO in models adjusted for age, sex, and CRP, whereas the evaluated hemogram‐derived inflammatory indices were not associated with RVO in models adjusted for age and sex. However, hypertension, diabetes mellitus, smoking status, body mass index, cardiovascular or renal disease, and lipid‐lowering treatment were unavailable and could not be incorporated into the analyses. The reported associations therefore cannot be considered independent of established vascular and metabolic risk factors. These findings should be regarded as exploratory and hypothesis‐generating and require confirmation in prospective studies with comprehensive vascular and metabolic phenotyping.

## Author Contributions

Nurcan Gürsoy conceived and designed the study, performed the statistical analysis, and drafted the manuscript. Şenay Deniz contributed to data collection and data acquisition.

## Funding

The authors received no specific funding for this work.

## Disclosure

All authors read and approved the final manuscript.

## Ethics Statement

Ethical approval was obtained from the Erzincan Binali Yıldırım University Non‐Interventional Clinical Research Ethics Committee (meeting no. 005, decision no. 2026–05/06, dated 05 March 2026). The study was conducted in accordance with the Declaration of Helsinki. Given the retrospective design and the use of anonymized routine clinical data, the requirement for informed consent was waived under local ethics procedures.

## Conflicts of Interest

The authors declare no conflicts of interest.

## Data Availability

The datasets generated and/or analyzed during the current study are not publicly available because they contain potentially identifiable clinical information, but are available from the corresponding author upon reasonable request and subject to institutional and ethical approval.
